# Thermally Activated Construction Waste as an Efficient Adsorbent for Free Fatty Acid Removal from Waste Cooking Oil

**DOI:** 10.1021/acsomega.6c03383

**Published:** 2026-09-12

**Authors:** Laís Fernanda Juchem do Nascimento, Joel Gustavo Teleken, Fabiano Bisinella Scheufele, Thompson Ricardo Weiser Meier, Paulo André Cremonez, Rodrigo Brackmann

**Affiliations:** † 646047 Federal University of Paraná, Department of Engineering, Palotina 85953-128, Brazil; ‡ 74354 Federal University of Technology − Paraná, Department of Bioprocess and Biotechnology Engineering, Toledo 85902-490, Brazil; § Federal University of Technology − Paraná, Department of Chemical Processes, Toledo 85902-490, Brazil; ∥ Federal University of Technology − Paraná, Department of Chemistry, Pato Branco 85503-390, Brazil

## Abstract

This study aimed to evaluate the reduction of free fatty acids (FFAs) in waste cooking oil (WCO) using calcined concrete, brick, and ceramic tile residues as adsorbents. Concrete, brick, and ceramic tile residues were collected, ground (590–297 μm), and calcined at 850 °C. Characterization using SEM-EDS, FTIR, XRD, and TGA revealed that the calcination process increased porosity and surface alkalinity, enhancing the presence of calcium oxide (CaO) and silicate (SiO_2_) phases, both active in acid neutralization. Among the tested materials, concrete waste showed the highest efficiency, achieving a 62% reduction in free fatty acids, compared to 23% for ceramic and 17.5% for brick residues. The kinetic data fit best to the Elovich model (R^2^ = 0.9865), indicating a chemisorption-controlled mechanism on heterogeneous active sites dominated by interactions between Ca^2+^ and carboxyl groups of fatty acids. The adsorption capacity reached 75 mg g^–1^, a value comparable to other alkaline adsorbents such as quicklime or calcium silicate, confirming the strong reactivity of concrete-derived materials. The FTIR spectra confirmed the formation of calcium carboxylates after adsorption, while XRD patterns showed crystalline transformations consistent with CaO and C–S–H phase reactivation. The pH increase from 7.5 to 10.5 during the adsorption process further confirmed the alkalinity of the adsorbent and the neutralization of FFAs. Comparative clustering analysis with literature data demonstrated that thermally treated concrete achieved high FFA reduction under mild operational conditions (25 °C, 150 rpm, 360 min), offering an energy-efficient alternative to conventional adsorbents. The results validate calcined concrete waste as a technically viable and environmentally sustainable adsorbent for the pretreatment of waste cooking oils.

## Graphical abstract

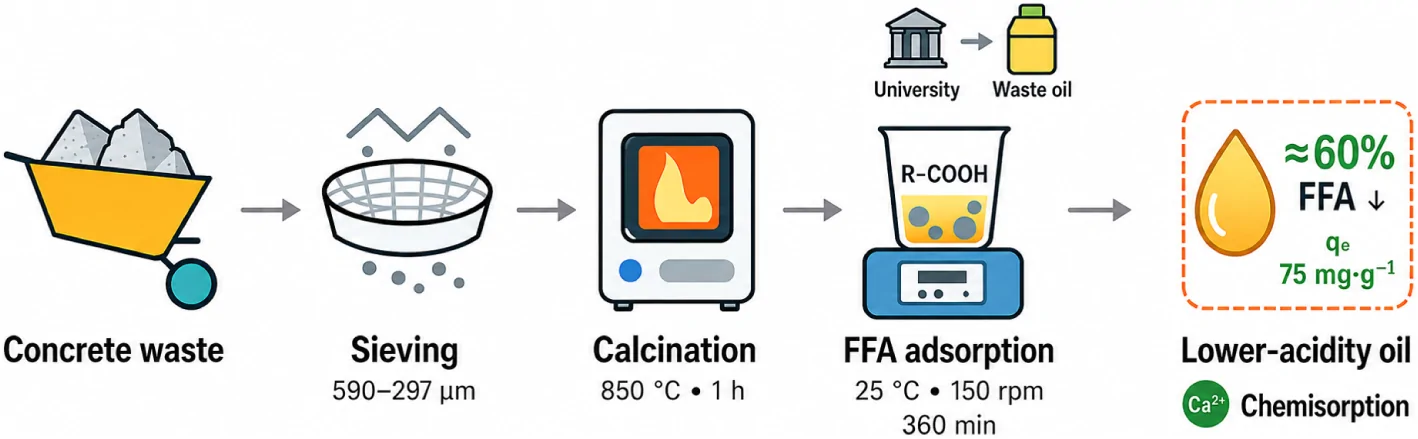

## 1 Introduction

The increasing generation of urban and industrial waste, coupled with the growing global demand for energy, has intensified the search for sustainable alternatives within the world’s energy matrix. The heavy reliance on fossil fuels, particularly mineral diesel, remains a major contributor to greenhouse gas emissions, thereby accelerating global warming and hindering progress toward the decarbonization goals established under the Paris Agreement. In this context, biodiesel, derived from vegetable oils and residual fats, has emerged as a renewable, biodegradable, and environmentally benign biofuel with physicochemical properties comparable to those of conventional diesel.^1^

Biodiesel production typically involves two main stages: esterification and transesterification of triglycerides with short-chain alcohols, such as methanol or ethanol, in the presence of homogeneous or heterogeneous catalysts. Despite the operational simplicity of these reactions, the economic feasibility of biodiesel synthesis remains constrained by the high cost of feedstock, which can account for up to 80% of the total production expenses.^2^

Against this backdrop, waste cooking oil (WCO) has emerged as a low-cost and widely available feedstock for biodiesel production. Its reutilization mitigates several environmental concerns, including the contamination of water bodies, the clogging of sewage networks, and the release of volatile organic compounds during decomposition. Furthermore, the valorization of WCO aligns with the principles of the circular economy, promoting the conversion of urban waste into clean and renewable energy sources.^3^

Nevertheless, a significant technical challenge associated with the use of waste cooking oil (WCO) is its high concentration of free fatty acids (FFAs). These compounds are formed through the hydrolysis and thermal oxidation of the oil during prolonged frying processes and are primarily responsible for soap formation when exposed to alkaline catalysts. This reaction hinders phase separation and substantially decreases the overall biodiesel yield. Studies have shown that the FFA content in WCO may range between 2% and 15%, depending on the frying duration and operating temperature.^4^

While biomass-based adsorbents are effective, inorganic waste-derived materials have also demonstrated promising potential for adsorption applications. In the context of waste cooking oil (WCO), to render it suitable for alkaline transesterification, it is essential to perform an acid-neutralization pretreatment that reduces the free fatty acid (FFA) content to levels below 2%. Adsorption techniques using these materials play a crucial role in achieving this reduction, enhancing the overall efficiency of the process. Several methods have been proposed for this purpose, including acid esterification, chemical neutralization, and, more recently, adsorption, which stands out as an efficient, cost-effective, and environmentally friendly approach. The adsorption process relies on the chemical and physical interactions between the oil and a solid adsorbent with high surface area, enabling the selective removal of FFAs and impurities.^5^ Studies have reported notable reductions in FFA levels using alternative and low-cost adsorbent materials. For instance,^6^ achieved a 93% reduction in FFA utilizing activated carbon derived from coconut fibers through a continuous adsorption process lasting 45 min.[Bibr ref7] (2021) Confirmed that activated carbon derived from banana peel exhibited Freundlich-type adsorption behavior, with a maximum adsorption capacity of 27.4 mg·g^–1^ of FFA removed. These findings highlight the predominance of physical adsorption mechanisms, emphasizing the effectiveness of lignocellulosic biomass-based adsorbents in reducing free fatty acid content in waste cooking oils.

Other inorganic waste-derived materials have also demonstrated remarkable performance as adsorbents.^8^ Reported that spent bleaching earth (SBE) reactivated with HNO_3_ reduced the acidity of waste cooking oil (WCO) by up to 85% after 90 min of contact, confirming its strong adsorption capability for free fatty acids. Similarly,^9^ demonstrated that fly ash activated with NaOH was able to remove up to 87% of FFAs, validating the potential of industrial residues as efficient and environmentally friendly adsorbents. In addition,^10^ found that calcium oxide (CaO) derived from hydrated lime achieved up to a 72% reduction in FFA content within 60 min, which underscores the potential of alkaline oxides as highly reactive adsorbents for FFA removal.

This performance is directly associated with the presence of alkaline and silicate compounds, a structural composition similar to that found in cementitious materials such as concrete. These scientific findings suggest that construction and demolition waste (CDW), particularly crushed concrete, holds substantial potential as an alternative adsorbent for the treatment of waste oils. The chemical composition of concrete, rich in CaO, SiO_2_, and Al_2_O_3_, closely resembles that of materials already recognized for their adsorptive efficiency and capacity to neutralize free fatty acids (FFAs). Moreover, the utilization of concrete waste contributes to reducing landfill disposal, mitigating environmental impacts, and valorizing construction residues, thereby aligning with the principles of the circular economy and sustainable energy production.

Accordingly, this study is based on the hypothesis that crushed concrete waste can act as an effective adsorbent for reducing the acidity of waste oils generated in university restaurants, thereby enabling their reuse as a viable feedstock for biodiesel production.

## 2 Materials and Methods

### 2.1 Description of Materials and Reagents Utilized throughout the Study

In this study, the materials used as adsorbents were residues obtained from an actual construction site and were separated into three categories: concrete, ceramic, and brick waste, each treated and analyzed individually. The adsorbate applied was used cooking oil collected from the university restaurant at UFPR (Federal University of Paraná).

The concrete waste used in this study was obtained from a conventional residential construction site in southern Brazil and is representative of ordinary Portland cement concrete commonly used in Brazilian civil construction. Based on XRD and EDS analyses, the material likely consisted of CP II-Z Portland cement, quartz sand, and crushed basalt aggregates, with an estimated 1:2:3 proportion (cement:sand:coarse aggregate). The predominance of quartz, calcite, and calcium-rich hydrated phases confirms a typical cementitious matrix suitable for alkaline activation and reactive adsorption after calcination.

The concrete was gathered directly from the construction site, then it was crushed manually and sieved using a set of standard laboratory sieves until particles within the range of 590 to 297 μm were obtained. After that, the material went through a heat treatment in a muffle furnace at 850 °C, for a period of 1 h. The heating rate used during this process was fixed at 10 °C min^–1^.^11^

### 2.2 Thermogravimetric Analysis (TGA)

Thermogravimetric analysis (TGA) was also performed to determine the samples’ weight loss as a function of temperature. The samples were heated under non-isothermal conditions, with a constant heating rate, to identify the temperature ranges corresponding to the highest mass loss. The three treatments concrete, ceramic, and brick waste were included in the analysis to compare their thermal behavior.

### 2.3 Energy-Dispersive X-ray Spectrometry

The main response evaluated was the reduction of free fatty acids, which was measured in triplicate. Based on these results, the subsequent analyses were carried out. To complement the microscopy analysis, the EDS (Energy Dispersive X-ray Spectroscopy, EDX, or EDS) tool was used, coupled with SEM (Scanning Electron Microscopy), as a semi-quantitative technique to determine the types and levels (in percentages) of chemical elements present in the tested portions of the adsorbents. The technique is based on energy-dispersive X-ray spectrometry. For this purpose, the Penta FET Precision equipment was employed.

### 2.4 Physicochemical Characterization

Before and after this thermal procedure, the residue that showed the best results was submitted for characterization to verify its physical and chemical properties. For that, SEM-EDS and FTIR (Fourier Transform Infrared Spectroscopy) analyses were carried out to observe changes in surface morphology, chemical composition, and structure. The SEM-EDS images, obtained from gold-covered samples using a high-resolution microscope, helped to visually identify the surface features and also the elements present. As for FTIR, the spectra were collected in the 4000 to 400 cm^–1^ region (using the KBr pellet method), allowing the detection of functional groups and any structural alterations that may have occurred after heating. The X-ray diffraction (XRD) analysis was performed using a Rigaku Miniflex 600 diffractometer. This equipment is equipped with a Cu Kα radiation source (λ = 0.154 nm), commonly used in XRD due to its suitable wavelength for crystalline material characterization. The system operates at a power of 600 W and a voltage of 40 kV, allowing for precise phase identification and structural analysis of crystalline samples.

### 2.5 Acidity Index Analysis

The adsorption experiments were carried out in batch mode using a Dubnoff water bath for agitation. Equilibrium tests were conducted in 250 mL Erlenmeyer flasks containing 33 g of waste cooking oil and 5 g of thermally treated mortar as the adsorbent. The residual oil collected from the university restaurant presented an acid value of 2.539 ± 0.31 mg KOH g^–1^. The acid value was calculated using eqs 1 and 2, as presented in Table 1, which describe the relationship between the volume of titrant used and the free fatty acid content in the oil. After centrifugation, the samples were analyzed for acid value following the ref 12. The adsorption capacity was calculated by the eq 3. Adsorption kinetics were performed with points collected at 5, 15, 30, 90, 120, 180, 300, and 360 min.

**Table 1:** Equations Used for the Calculation of Acid Value^a^

Equations	Parameters
1 $AV=\frac{V\times C_{KOH}\times M}{m}$	AV = Acid value (mg KOH·g^–1^ of oil); V = Volume of KOH relative used in the titration (mL); C = Concentration of the kOH solution (mol L^–1^); M = Molar mass of NaOH (g mol^–1^); m = Mass of oil (g).
2 $\%R=\frac{I_{Ai}-I_{Af}}{I_{Ai}}\times 100$	%R = Percentage of acid value reduction.
3 $q_{t}=\frac{(AV_{0}-AV_{t})m_{oil}M_{FFA}}{M_{KOH}m_{ads}}$	*where AV*_0_ and *AV*_*t*_ are the initial acid value and the acid value at time t; *m_oil_* is the mass of oil; *m_ads_* is the mass of adsorbent; and *M_FFA_* and *M_KOH_* are the molar masses of the reference FFA and KOH, respectively.

a Note: Values are presented as mean ± SD (n = 3).

The acidity index was calculated in mg NaOH g^–1^ of the sample (eq 1). To convert this value to milligrams of potassium hydroxide (KOH), the result was multiplied by 1.4, since KOH has 1.4 times the molar mass of NaOH.

### 2.6 Analysis of Adsorption Mechanism: Kinetics

The treatment that showed the best average performance was selected for kinetic pH analysis. The experimental data were fitted to nonlinear forms of the pseudo-first-order (PFO) and pseudo-second-order (PSO) models. The respective equations are presented below (Table 2).

**Table 2:** Equations Used for Adsorption Capacity and Kinetic Modeling

Equations	Parameters
PFO: 4 $q_{t}=q_{e}\left(1-e^{-k_{1}t}\right)$	q_e_ and q_t_ (mg g^–1^) are the amounts adsorbed at equilibrium and at time t, respectively. *k*_1_ (min^–1^) is the rate constant of the pseudo-first-order model.
PSO: 5 $q_{t}=\frac{k_{2}q_{et}^{2}}{\left(1+k_{2}q_{e}t\right)}$	*k*_2_ (g mg^–1^ min^–1^) is the rate constant of the pseudo-second-order model.
Elovich: 6 $q_{t}=\frac{1}{\beta }\ln\left(\alpha \beta \right)+\frac{1}{\beta }\ln\left(t\right)$	α (mg g^–1^ min^–1^) is the initial adsorption rate, and β (g mg^–1^) is related to surface coverage and activation energy.

To compare the application of different kinetic models, nonlinear regression was applied using the least squares method, and the quality of the fit was evaluated using the coefficient of determination (R^2^) and the sum of squared errors (SSE). The initial parameters for each kinetic model were estimated from the experimental curves and refined by least-squares minimization until convergence was achieved.

### 2.7 Steps of the Methodology

This diagram (Figure 1) outlines the methodological steps employed to evaluate the use of construction waste as adsorbents for reducing free fatty acid (FFA) levels in waste cooking oil. Three types of residues, concrete, brick, and ceramic were collected from construction sites, ground, sieved (590–297 μm), and thermally treated at 850 °C for 1 h. The waste oil, obtained from a university restaurant, was pretreated by mixing 5 g of residue with 33 g of oil under agitation (150 rpm, 25 °C) for 360 min in a Dubnoff bath. After centrifugation, the samples were analyzed for acid value.^12^ Residues with the best adsorption capacity (mg·g^–1^) were further evaluated through kinetic to support the final selection of the most efficient adsorbent.

**Figure 1:**
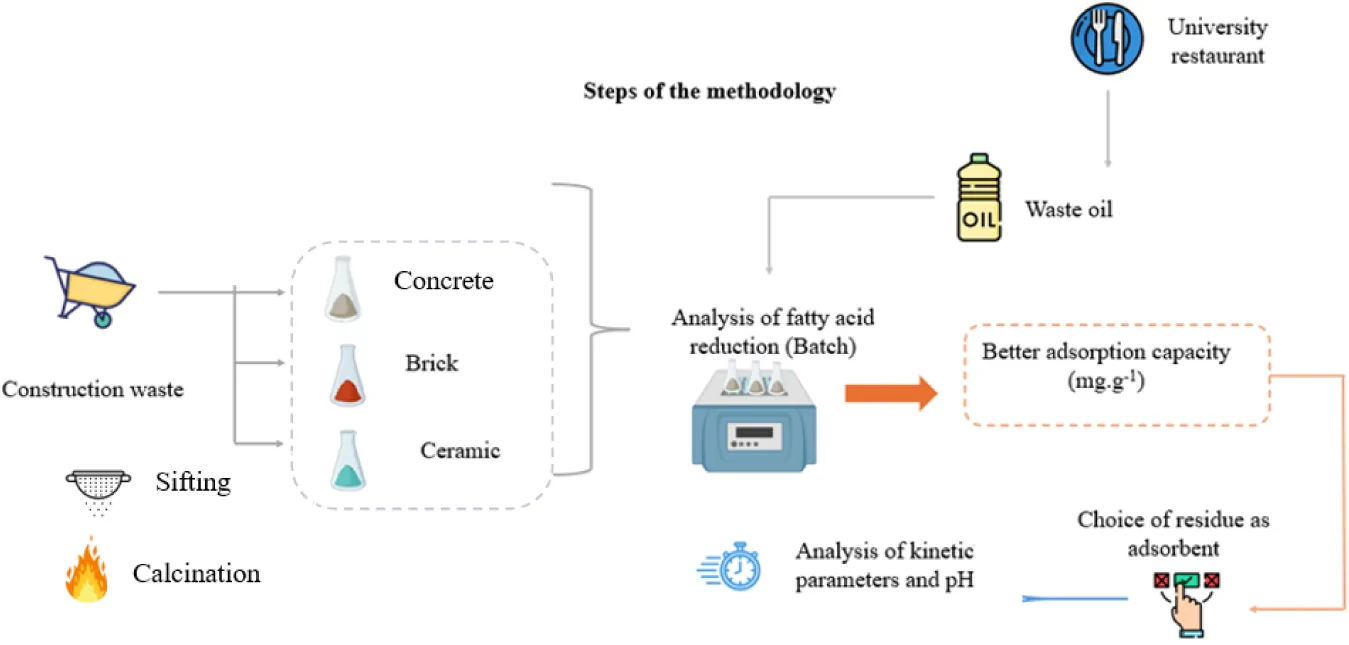
Methodological framework for adsorptive pretreatment using construction waste.

Finally, the performance of different adsorbent and catalyst materials reported in the literature was compared with the materials developed in this study. The variables considered were adsorption temperature, reduction of free fatty acids (FFA), and reaction time. These variables were normalized to ensure comparability between studies using different scales and experimental conditions. Normalization was performed using the min-max scale, converting the data to a range between 0 and 1. The resulting heat maps were generated using OriginPro software (OriginLab Corporation, USA). The color gradient in the heat map represents the normalized values, ranging from 0 (light colors) to 1 (dark colors). Each row corresponds to a different material or catalyst, while each column represents one of the parameters studied (Adsorption_Temp_norm, Reduction_FFA_norm, and Time_norm).

## 3 Results and Discussion

### 3.1 Comparative Performance of Adsorbents in Free Fatty Acid Reduction

The data presented in Table 1 illustrate how different materials subjected to thermal treatment at 850 °C influence the removal of free fatty acids (FFAs) in waste cooking oils. Among the tested materials, CONC exhibited the highest performance, achieving a 62.00% reduction in impurities. In contrast, the TILE and BRICK samples showed lower efficiencies, with reductions of 23.00% and 17.50%, respectively.

The data in Figure 2 demonstrate that concrete was the most efficient material for removing free fatty acids (FFA), a result that can be explained by the significant presence of calcium oxides (CaO) and hydrated calcium silicates (C–S–H) in its composition. These components confer high alkalinity and chemical neutralization capacity, favoring the formation of calcium salts (carboxylates) through acid–base reactions between the carboxylic groups (−COOH) of the FFA and the Ca^2+^ ions present on the material’s surface. Furthermore, the porous and irregular morphology of concrete, described,^13^ increases the surface area of contact and the diffusion of acid molecules to the active sites, which enhances the adsorptive process and the occurrence of neutralization.

**Figure 2:**
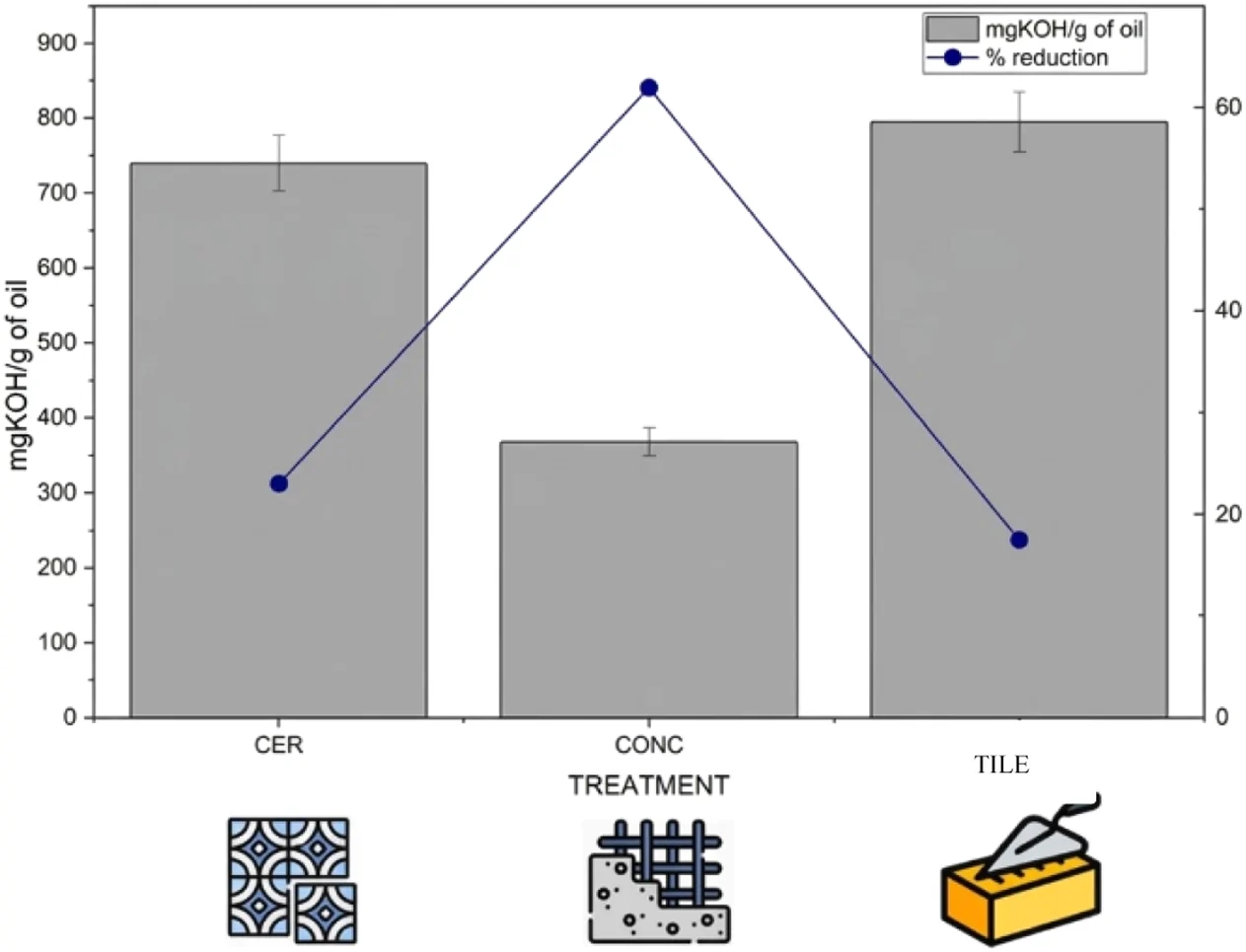
Treatments with different thermally treated materials (concrete, brick, and ceramic) and their respective acid value (FFA) reduction efficiencies per gram of waste oil.

Ceramic residues, composed predominantly of SiO_2_ and Al_2_O_3_ in crystalline form, exhibit acidic character and lower chemical reactivity, limiting adsorption to van der Waals physical interactions and surface forces, which explains their reduced efficiencies. This difference was also highlighted by,^14^ who demonstrated that layered double hydroxides (LDH) and anionic clays possess adjustable basic sites (Bronsted–Lewis).

The percentage reduction values presented in Table 1 and Figure 2 correspond to the decrease in the acid value of the waste cooking oil after adsorption treatment. In contrast, the adsorption capacity (q) expresses the equivalent mass of free fatty acids removed per gram of adsorbent during the kinetic experiments. The equilibrium adsorption capacity obtained from the pseudo-second-order kinetic model reached 75.18 mg g^–1^, indicating that each gram of calcined concrete was capable of removing approximately 75 mg of FFA-equivalent compounds under the evaluated conditions. This distinction was added to avoid ambiguity between the overall acid value reduction (%) and the adsorption capacity derived from kinetic modeling.

The markedly different performances of the construction residues suggest that FFA removal was governed by both mineral surface chemistry and pore accessibility. In calcined concrete, thermal activation exposed basic CaO, MgO, and calcium-silicate sites, at which the carboxylic head groups of FFAs may undergo deprotonation and coordinate with surface Ca^2+^ or Mg^2+^ ions, forming mono- or bidentate surface complexes and calcium/magnesium carboxylates. Similar carboxylate–metal interactions have been reported for calcium silicate, CaO, and other mineral oxide surfaces.^34,38–41^ This interpretation is consistent with the appearance of aliphatic C–H bands and the changes observed in the carboxylate region of the FTIR spectra, as well as with the Elovich kinetic behavior, although kinetic fitting alone does not conclusively demonstrate chemisorption. In contrast, brick and ceramic tile residues, composed predominantly of relatively inert crystalline SiO_2_–Al_2_O_3_ phases and containing fewer accessible basic cationic sites, likely retained FFAs mainly through pore filling, hydrogen bonding with surface hydroxyl groups, and weak dispersion forces, explaining their lower removal efficiencies. Hydrophobic interactions may contribute after the initial anchoring of the polar carboxylic groups, as the aliphatic chains can associate laterally and form an oil-wet organic layer. Nevertheless, because the adsorption occurred in a predominantly nonpolar oil phase, these contributions are more accurately described as chain–chain van der Waals interactions rather than a classical water-driven hydrophobic effect. Therefore, acid–base reaction and carboxylate complexation appear to be the dominant mechanisms for calcined concrete.

### 3.2 Thermal Characterization (TGA/DTG) of Construction Waste

As shown in Figure 3, the TGA and DTG curves reveal distinct differences in the thermal decomposition behavior of the concrete, brick, and ceramic tile residues, reflecting variations in their composition and thermal stability.

**Figure 3:**
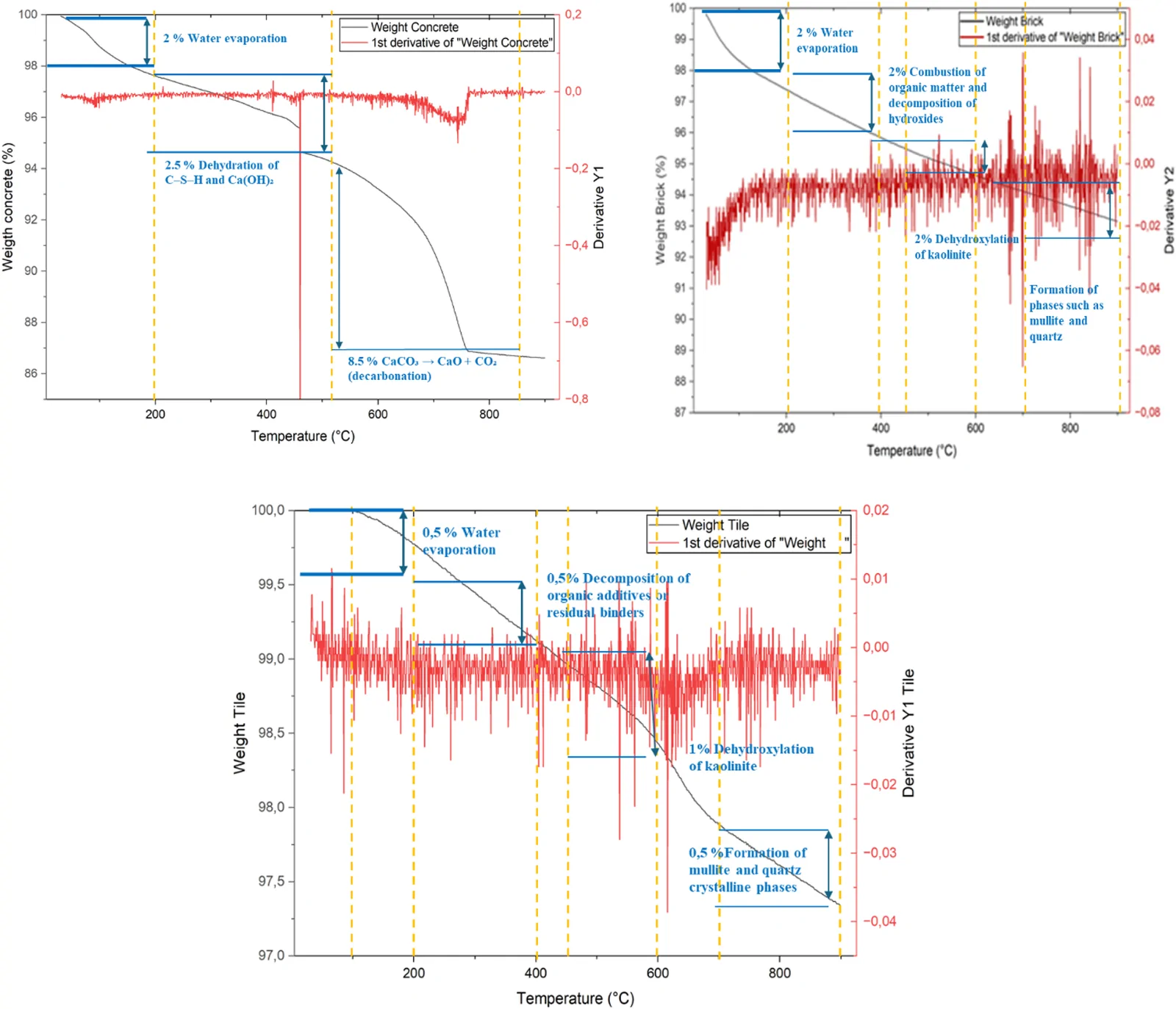
Thermogravimetric (TGA) and differential thermogravimetric (DTG) curves of ceramic tile residue showing high thermal stability and low overall mass loss.

The thermogravimetric (TGA/DTG) analysis of the construction residues revealed distinct multi-step mass-loss profiles reflecting their mineralogical composition and prior thermal exposure. Concrete presented three main decomposition stages: dehydration below 200 °C (loss of adsorbed water), dehydroxylation between 400–550 °C (Ca­(OH)_2_ → CaO), and decarbonation above 650 °C (CaCO_3_ → CaO + CO_2_), consistent with observations for cementitious materials reported by^10^ Brick and ceramic tile samples exhibited smoother profiles with minor total mass losses (<5%) and thermal events between 400–700 °C associated with aluminosilicate dehydroxylation and carbonate decomposition, as reported.^9^ The higher thermal stability of brick and tile residues indicates a sintered, inert matrix, whereas the alkaline composition of concrete waste—with active CaO and silicate phases—suggests greater potential for adsorptive and catalytic applications in the pretreatment of waste cooking oils for free fatty acid reduction.^7,8^

### 3.3 Morphological Analysis by Scanning Electron Microscopy (SEM)

A control experiment using non-therally treated concrete was performed; however, the material exhibited no significant FFA adsorption or acid value reduction compared to the calcined sample. This result confirms that the thermal activation at 850 °C was essential to enhance surface reactivity and generate active alkaline phases responsible for the adsorption/neutralization process (Figure 4).

**Figure 4:**
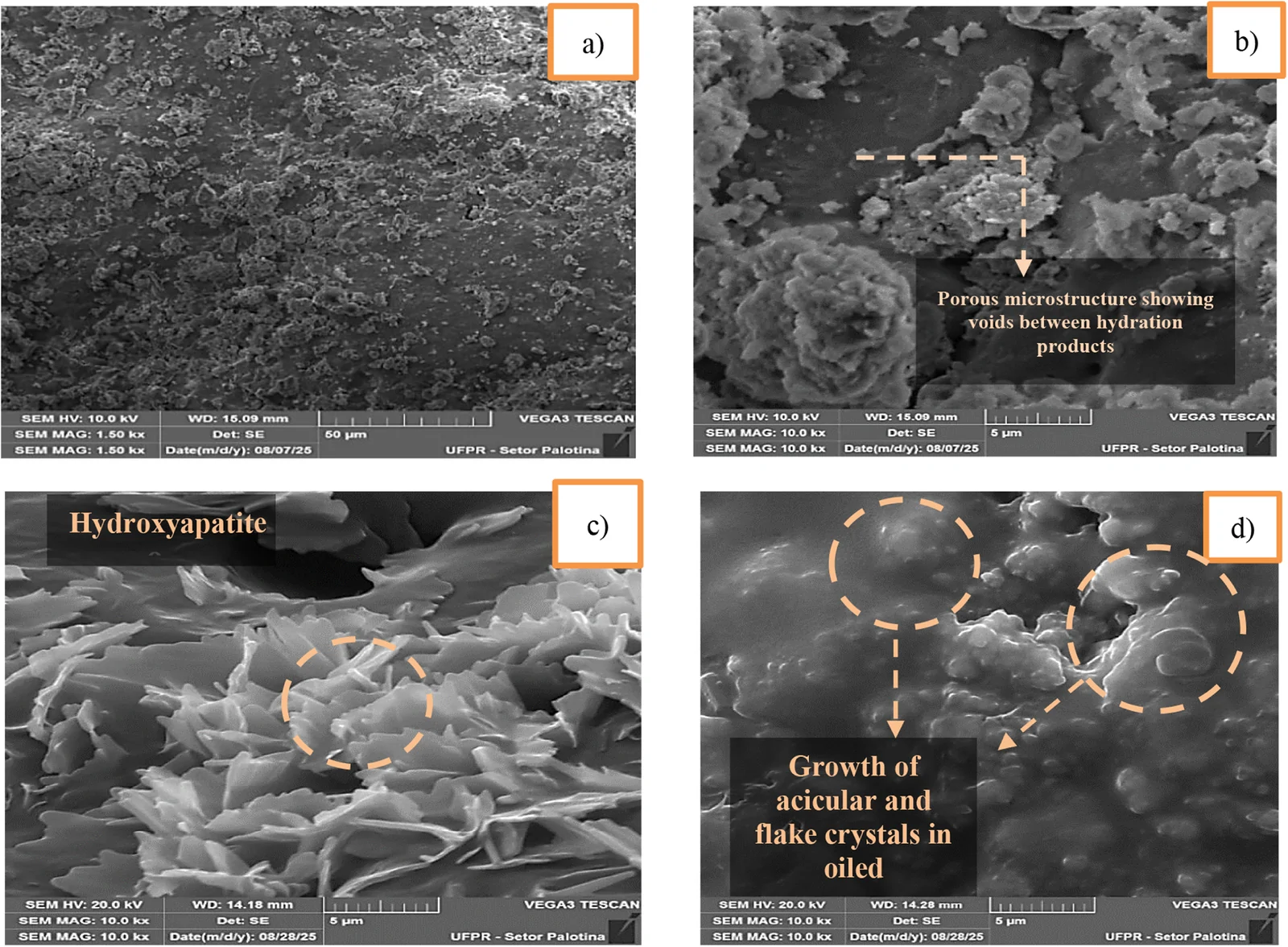
SEM micrographs of modified mortar surface: (a) and (b) thermally treated at 850 °C; (c) heat-treated at 850 °C after FFA adsorption and (d) heat-treated at 850 °C after FFA of adsorption.

Thermal treatment of concrete powder at temperatures close to 800 °C enhances the release of Ca^2+^ ions, promoting the formation of a calcium-rich surface layer and facilitating the precipitation of calcium phosphates, provided that the concrete or oil contains significant amounts of phosphorus, as reported by ref 15, 16 . Within this temperature range, aluminosilicate phases exhibit limited reactivity with Ca­(OH)_2_ due to CO_2_ release during CaCO_3_ decomposition, which contributes to the development of a more porous structure while preserving the integrity of the silicoaluminate framework.

Silicon (Si) and aluminum (Al), mainly associated with C–S–H and C–A–S–H phases, remain structurally stable after heating, indicating a structural rearrangement rather than a complete breakdown of the aluminosilicate network. Calcium, initially present predominantly as hydroxide or carbonate, is converted into CaO during thermal activation, increasing Ca^2+^ availability and surface reactivity, which are essential for interactions with acidic species such as free fatty acids. Moreover, cementitious matrices exhibit excellent thermal stability at temperatures up to 900 °C, as demonstrated by^17,18^ also observed similar formations, such as the development of acicular and flake-like crystal structures in lime–brick mortars treated with oil-based additives. These formations contributed to an enhanced microstructural consistency. Oil-treated cementitious matrices exhibited significantly improved surface durability when compared to control samples.^19^

### 3.4 Chemical Composition of Concrete

The presented data confirm the presence of calcium, resulting from the addition of quicklime to the conventional mortar mixture prepared in situ. The main component identified is C, O and H, accounting for 62.50% of the mass, followed by calcium at 58.80% and 90.80% in the mortar containing oil (Figure 5).

**Figure 5:**
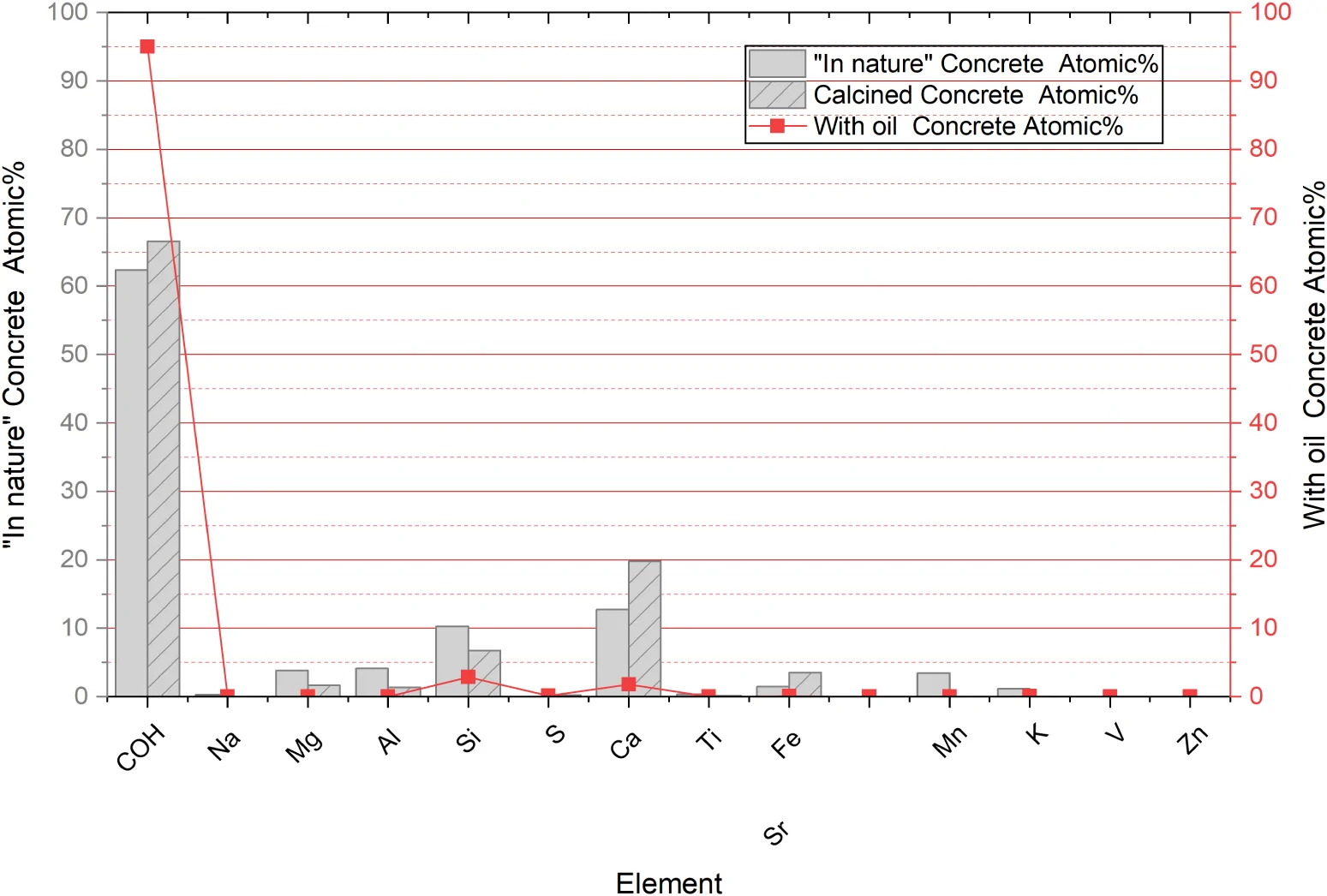
Elemental composition (atomic %) of concrete samples: in nature, calcined, and with oil.

The elemental analysis highlights the essential role of Ca, Mg, Al, and K in the adsorption and neutralization of free fatty acids (FFAs). After calcination, a significant increase in Ca (25.35%) and Mg (6.15%) is observed, confirming the formation of highly active CaO and MgO phases. These phases provide strong basic sites capable of neutralizing FFAs through ionic interactions, as widely reported for calcined CaCO_3_-rich materials and MgO-based adsorbents.^20^

### 3.5 Thermal Effects on Functional Groups and Crystalline Phases of Concrete Waste: An FTIR-XRD Comparative Approach

FTIR spectroscopic analysis (Figure 6) allowed the identification of the main differences among three types of mortar: natural concrete, calcined concrete, and oil-modified concrete. The interpretation was based on typical wavenumber ranges associated with functional groups found in both inorganic and organic materials.

**Figure 6:**
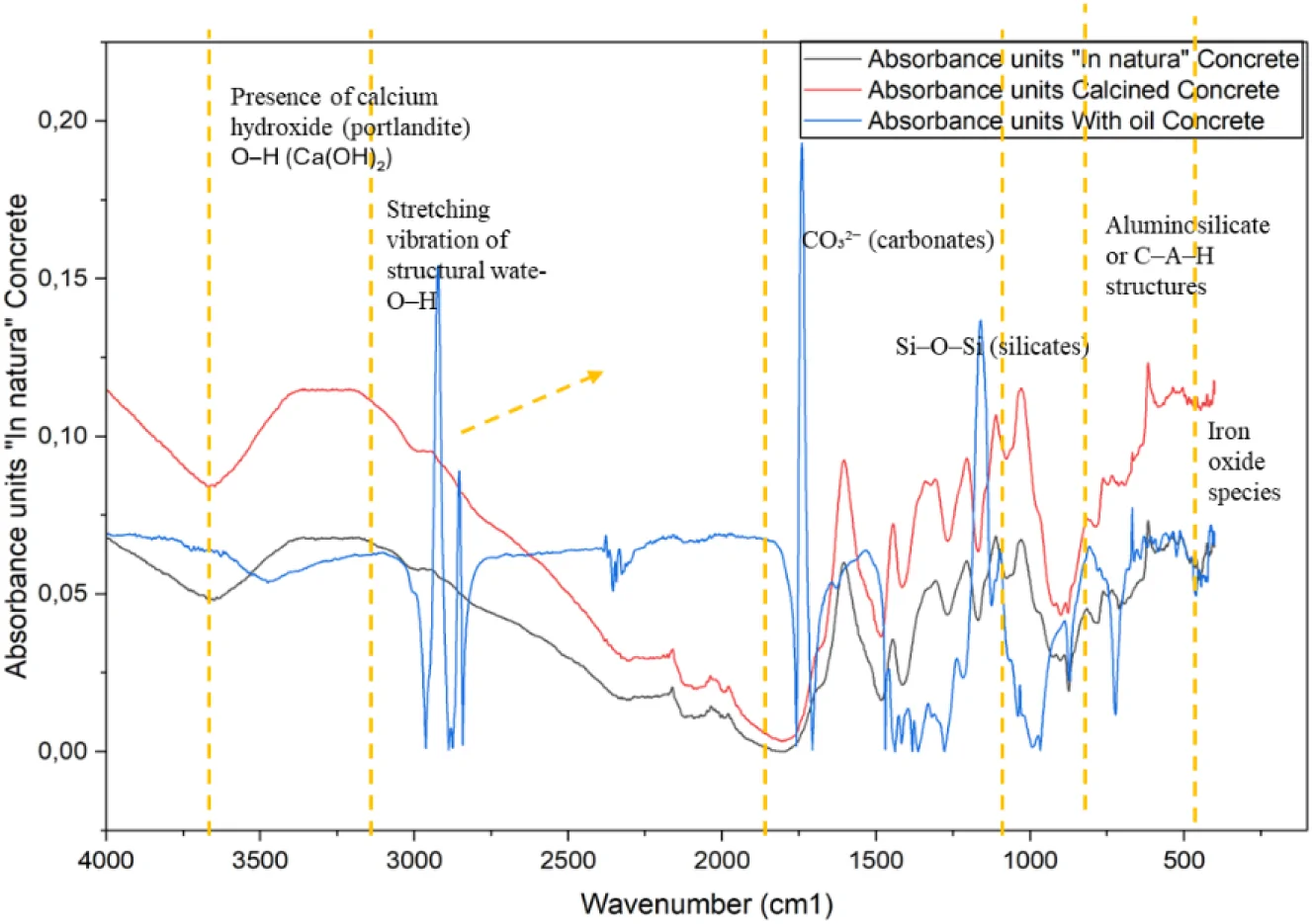
Comparative FTIR spectra of concretes: thermally treated at 850 °C; thermally treated at 850 °C after adsorption of FFA.

The FTIR spectrum of the oil-modified mortar revealed several significant changes compared to the untreated and calcined samples. The appearance of new absorption bands between 2850–2950 cm^–1^ corresponds to the C–H stretching vibrations of aliphatic fatty acid chains, confirming the incorporation of organic compounds from the oil into the cementitious matrix. This is consistent with previous results that identified fatty acid residues in lime mortars and related them to the hydrophobic behavior of historical materials.^21^

A sharp reduction in the O–H stretching region (3640–3400 cm^–1^) suggests a decrease in hydroxyl groups and bound water, indicating that the oil acted as a hydrophobic agent. Similar observations were made when linseed oil was incorporated into lime-based systems, where it hindered carbonation and decreased water absorption by forming an organic coating on the pore surfaces.^22^ In the carbonate region (1400–1500 cm^–1^), slight shifts and broadening of the peaks may indicate the formation of calcium carboxylates (metallic soaps) due to the interaction between the fatty acids of the oil and the calcium ions of the binder. This chemical reaction, also observed by,^23^ results in hydrophobic films that reduce capillary absorption and improve moisture resistance. Studies that used hydrophobic agents based on vegetable oil reported significant reductions in mass loss and water permeability in treated mortars, confirming that fatty acid–based coatings create a durable barrier against water ingress while maintaining partial breathability.^24^ After adsorption, the bands at approximately 1540–1650 and 1400 cm^–1^ were assigned to the asymmetric and symmetric stretching vibrations of COO^–^, respectively, indicating the formation of surface carboxylate species through interactions between FFAs and calcium-rich sites. Variations in the Si–O–Si stretching band near 1000 cm^–1^ may be related to surface coverage and changes in the silicate environment after oil adsorption. These results are consistent with, calcium carboxylate formation.

The XRD profile of the oil-contacted concrete (Figure 7c) exhibited pronounced diffuse scattering and partial peak overlap, particularly in the approximately 15–26° 2θ region, superimposed on the remaining crystalline reflections. In the absence of quantitative pattern normalization, instrumental-broadening correction, and peak-width analysis, this feature should not be interpreted as direct evidence of crystallite-size reduction, bulk structural collapse, or FFA-induced destruction of the C–S–H phase. Long-chain fatty acids can react with calcium-rich mineral surfaces to form insoluble calcium soaps. In limestone-containing cement mortars, oleate species have been reported to react with Ca-rich sites and form calcium oleate coatings.^43^ Studies involving fatty-acid-modified calcium carbonate further demonstrated that these surface layers may contain both chemisorbed calcium carboxylates and physically adsorbed or multilayer organic material.^44^ Moreover, oleate adsorption can influence the crystal structure and morphology of CaCO_3_ and promote the precipitation of calcium oleate aggregates.^45^ Therefore, the diffuse region observed in Figure 7c is tentatively attributed to a poorly ordered organic/calcium-carboxylate interphase, potentially containing calcium oleate, together with residual oil covering the particle surfaces. Organic surface coverage may attenuate the diffraction signal and increase the diffuse background; however, it does not, by itself, demonstrate destruction of the underlying crystalline phases. The chemical contribution to this interphase is supported by the COO^–^ stretching bands detected by FTIR at approximately 1540–1650 and 1400 cm^–1^. Furthermore, C–S–H is intrinsically poorly crystalline, and significant dehydration, shrinkage, and phase reorganization are expected when it is exposed to temperatures approaching or exceeding 800 °C.^46^ Consequently, part of the structural modification of the calcium-silicate phases had already occurred during calcination at 850 °C, before contact with the oil. The present XRD and FTIR results therefore support a local reaction between FFAs and Ca-rich surface sites, accompanied by organic surface coverage, whereas additional bulk collapse of the calcium-silicate matrix after oil adsorption was not demonstrated. Confirmation of crystallite-size reduction or quantitative amorphization would require normalized XRD patterns acquired under identical conditions, peak deconvolution, instrumental-broadening correction, and quantitative phase analysis, such as Rietveld refinement with an internal standard.

**Figure 7:**
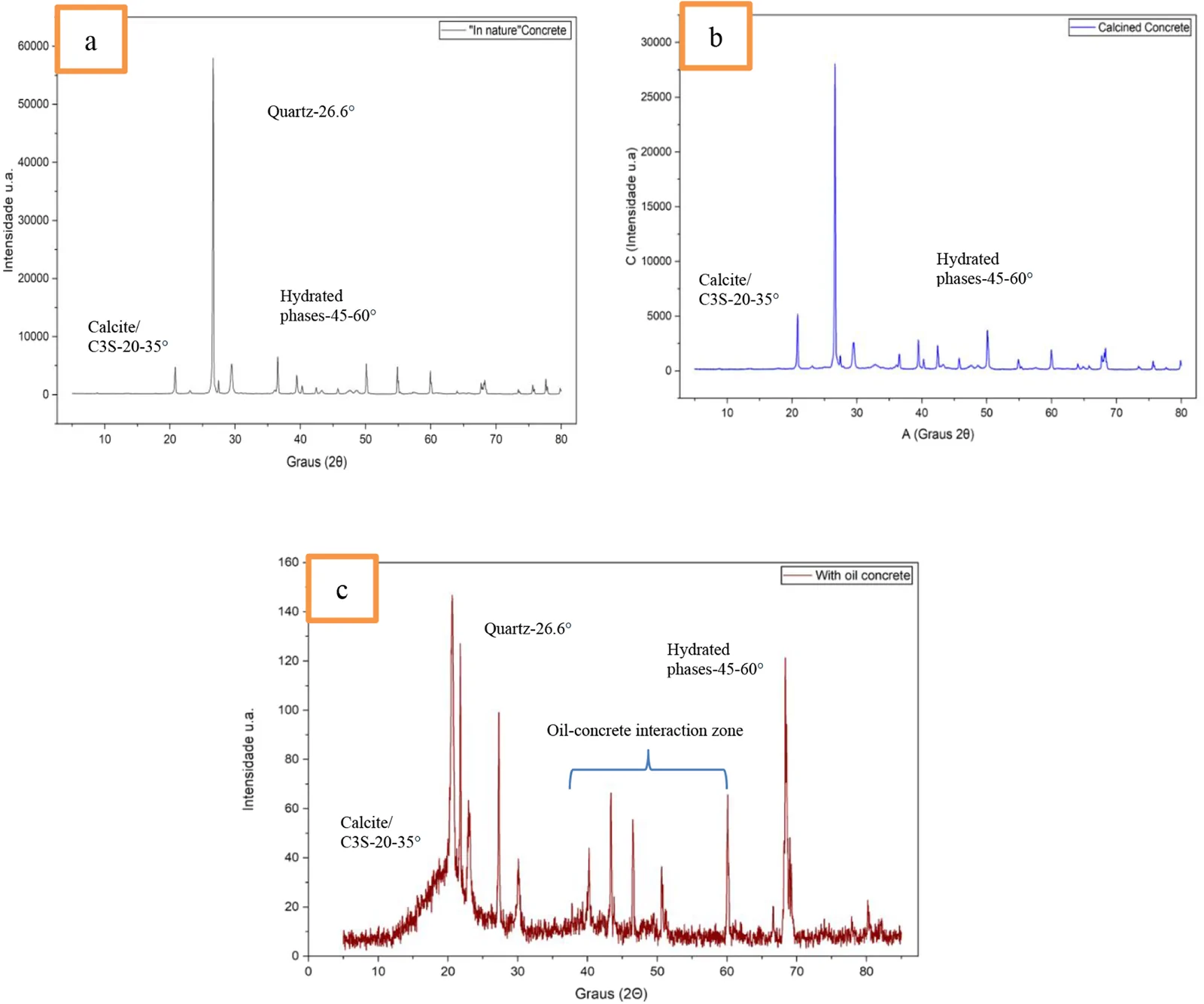
XRD patterns of (a) untreated concrete, (b) concrete calcined at 850 °C, and (c) calcined concrete after contact with waste cooking oil. The diffuse region observed in panel (c) is tentatively assigned to a poorly ordered organic/calcium-carboxylate interphase and residual organic surface coverage; it does not represent the identification of a discrete crystalline phase.

The increase in pH observed in the figure, from approximately 7.5 to 10.5, is associated with the neutralization of free fatty acids (FFAs) by the alkaline mineral matrix of the concrete. Similar behavior has been reported in systems using natural adsorbents such as activated zeolite^25,26^ and bentonite clay,^27^ which act as weak bases capable of adsorbing carboxylic compounds, thereby increasing the medium’s pH.

The gradual reduction of FFAs over time, reaching about 60% after 6 h, follows a typical adsorption equilibrium between internal diffusion and surface interaction. Similar trends have been observed with cocoa shell and zeolite adsorbents, showing significant FFA reduction within 60–90 min of contact.^28,29^

The pH was measured exclusively in an aqueous suspension of the adsorbent prepared with distilled water, and not directly in the oil phase. This procedure was adopted to evaluate the intrinsic alkalinity of the thermally activated concrete after calcination and its behavior over time (Figure 8).The pH was measured exclusively in an aqueous suspension of the adsorbent prepared with distilled water, rather than directly in the oil phase. This procedure was adopted to evaluate the intrinsic alkalinity of the thermally activated concrete after calcination and its behavior over time, as shown in Figure 8.

**Figure 8:**
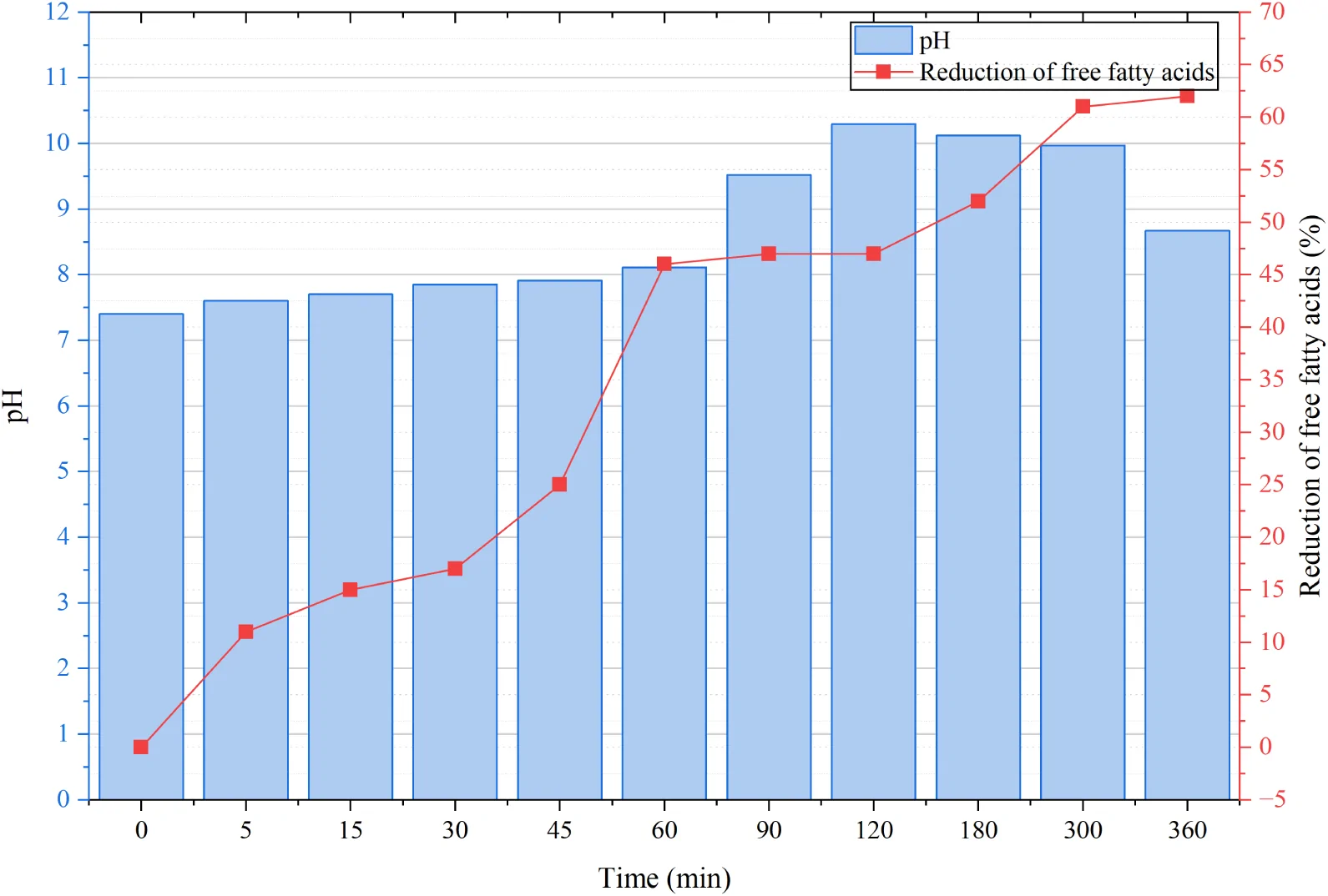
Variation of pH and reduction of free fatty acids over time using concrete as adsorbent.

Mineral adsorbents such as bentonite, zeolite, and activated fly ash show performance comparable to concrete due to their alkaline oxides (CaO, SiO_2_, Al_2_O_3_), which promote acid neutralization.^9^ Research also indicates that FFA removal efficiency can exceed 70% when thermally activated adsorbents are used.^31^ Contact time and temperature significantly affect adsorption rates. Studies with palm oil^32^ and used cooking oil^33^ demonstrate that longer reaction times and moderate temperatures (60–80 °C) enhance adsorption efficiency, while the pH increase reflects the progressive neutralization of FFAs.

### 3.6 Mechanistic Insights from Adsorption Kinetics

The experimental data of free fatty acid (FFA) adsorption capacity (qt) onto the calcined residue surface are presented in Figure 9, where the results were fitted by four classic kinetic models: Elovich, pseudo-first-order (PFO) and pseudo-second-order (PSO). The models: Elovich provided the best correlation with the experimental data, indicating that the rate-limiting step is predominantly governed by chemisorption.

**Figure 9:**
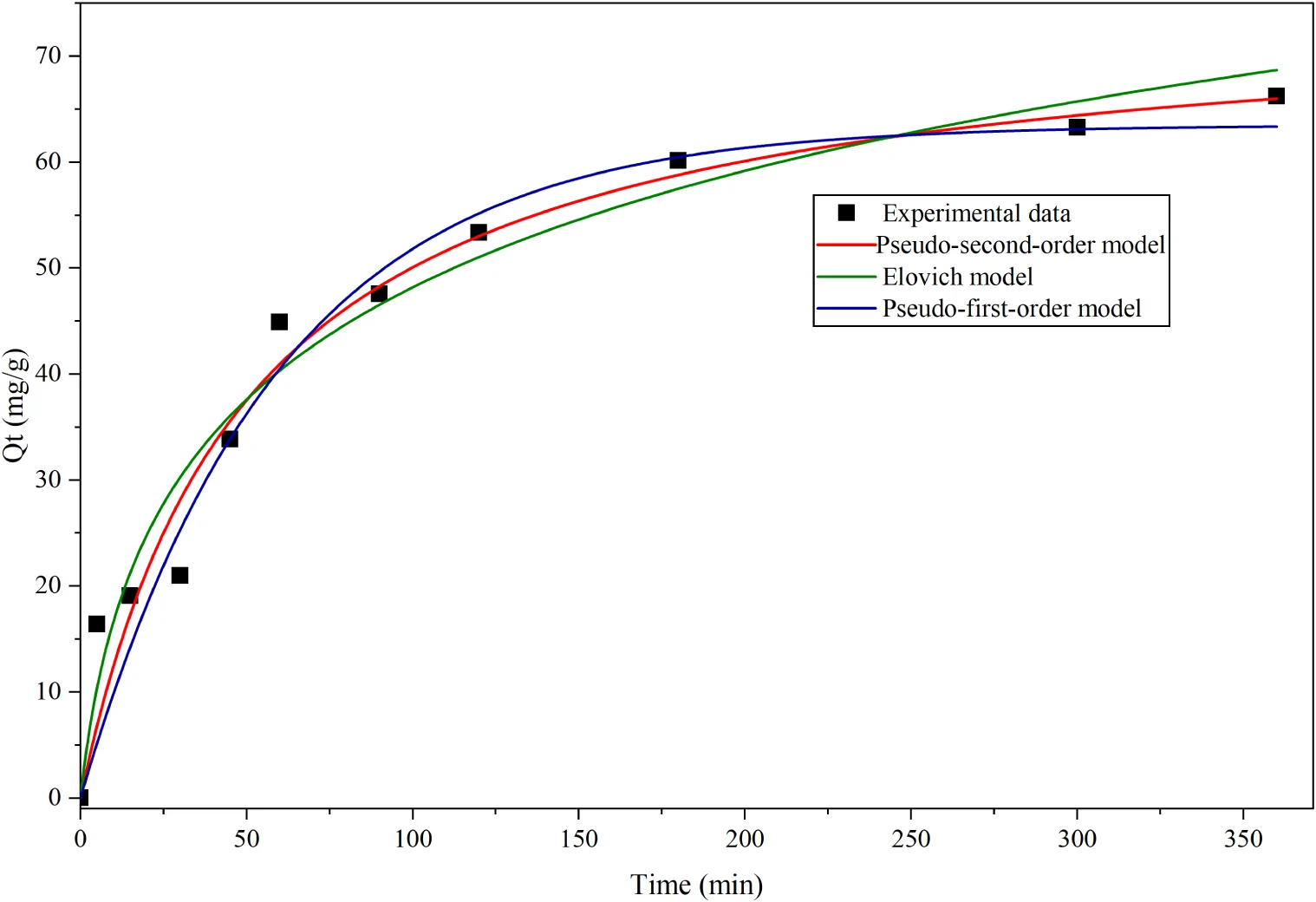
Adsorption kinetics of free fatty acids using concrete as adsorbent: comparison of pseudo-first-order, pseudo-second-order, and Elovich models.

The kinetic results obtained in this study demonstrate that the adsorption of free fatty acids (FFAs) by thermally treated concrete at 850 °C is best described by the Elovich model (R^2^ = 0.9865), indicating a process governed by chemisorption on heterogeneous surfaces. This behavior is attributed to the mineral composition of the adsorbent, mainly CaO, SiO_2_, and Al_2_O_3_, which provides multiple active sites with variable activation energies. Comparable findings were reported by,^34^ who applied calcium silicate derived from rice husk ash and observed that the Elovich model accurately represented the adsorption of FFAs due to surface heterogeneity and the formation of stable complexes between carboxylic groups and Ca^2+^ ions. Similarly,^10^ demonstrated that quicklime (CaO) followed a pseudo-second-order kinetic model, driven by chemical interactions between FFAs and Ca^2+^ reactive sites, achieving a 72% reduction of FFAs within 1 h.

Although the Elovich model indicated that chemisorption predominates during the adsorption process, the contribution of physisorption mechanisms should also be considered, particularly during the initial stages of contact. The porous and irregular morphology generated after calcination, as evidenced by the SEM micrographs, likely facilitates the rapid diffusion and temporary retention of free fatty acid (FFA) molecules through weak intermolecular interactions, including van der Waals forces and pore-filling effects. Similar synergistic adsorption pathways involving initial physisorption followed by chemically controlled adsorption have been reported for porous calcium-based and silicate adsorbents applied in waste oil treatment.^39,40^

As the adsorption process progresses, alkaline active sites such as CaO and partially reactivated calcium silicate phases promote stronger acid–base interactions with the carboxylic groups of FFAs, leading to the formation of calcium carboxylates and heterogeneous chemisorption behavior. This interpretation is supported by the FTIR spectra, which revealed characteristic COO^–^ stretching vibrations after adsorption, as well as by the superior fitting of the Elovich kinetic model, commonly associated with heterogeneous chemisorption processes occurring on energetically non-uniform surfaces.^41,42^ Therefore, the adsorption mechanism can be more accurately described as a synergistic process involving an initial physisorption stage followed by dominant chemisorption and surface neutralization near equilibrium (Table 3). Therefore, the adsorption mechanism can be more accurately described as a synergistic process involving an initial physisorption stage, followed by dominant chemisorption and surface neutralization as the system approaches equilibrium, as summarized in Table 3.

**Table 3:** Kinetic Modeling Parameters for FFA Adsorption of Thermally Treated Mortar at 850 °C: Comparison of PSO, PPO and Elovich, Models

Model	Parameter 1	Parameter 2	Mean error (mg g^–1^)	R^2^	Adjusted R^2^
Pseudo-second-order (PSO)	*k*_2_ (min^–1^) = 2.65216 × 10^–4^ ± 6.40561 × 10^–5^	q_e_ = 75.18246 ± 4.43045 (mg g^–1^)	—	0.96558	0.96176
Elovich	α = 2.88769 ± 0.04154	β = 0.06053 ± 3.40936 × 10^–4^	—	0.98650	0.98648
Pseudo-fisrt-order (PSO)	a = 63.49371 ± 0.18997	b = 0.01694 ± 2.0525 × 10^–4^	—	0.95141	0.95136

In terms of performance, the adsorption capacity of calcined concrete (75 mg g^–1^) was intermediate among reference materials, substantially higher than the banana peel activated carbon studied by^7^ (27.4 mg g^–1^, Freundlich model, physisorption) and lower than that of,^34^ who achieved an adsorption capacity of 5.67 mmol g^–1^ using highly pure calcium silicate under optimized conditions. Despite its lower maximum capacity, the concrete-based adsorbent displayed kinetic behavior comparable to CaO and calcium silicate systems, confirming that chemisorption predominates over diffusion-controlled mechanisms. These findings substantiate the potential of calcined concrete waste as a technically viable, low-cost, and sustainable adsorbent for pretreatment of waste cooking oils, promoting both acid value reduction and circular reuse of construction residues in biodiesel production processes.

### 3.7 Hierarchical Clustering Analysis of Adsorption Temperature, Free Fatty Acid Reduction, and Contact Time

The hierarchical clustering heatmap based on normalized adsorption temperature, free fatty acid (FFA) reduction, and contact time reveals distinct performance patterns among the evaluated adsorbents. Materials such as bentonite clay, eggshell-derived CaO, sengon sawdust biochar, and NaOH-activated water hyacinth clustered in regions characterized by high normalized FFA reduction values (≥0.75), albeit often associated with elevated temperatures and extended contact times. These results corroborate previous studies reporting that strong basic or alkaline surfaces enhance FFA removal but frequently require harsher operational conditions to achieve equilibrium, increasing energetic and operational demands.^35,37,38^ In contrast, polymeric and silica-based systems, including polysulfide polymers and silica gel, exhibited moderate FFA reduction combined with lower normalized temperature values, indicating a predominance of physical adsorption mechanisms with limited acid–base contribution^36^ (Figure 10). In contrast, polymeric and silica-based systems, including polysulfide polymers and silica gel, exhibited moderate FFA removal at lower normalized temperatures, suggesting the predominance of physical adsorption mechanisms with a limited contribution from acid–base interactions, as shown in Figure 10.

**Figure 10:**
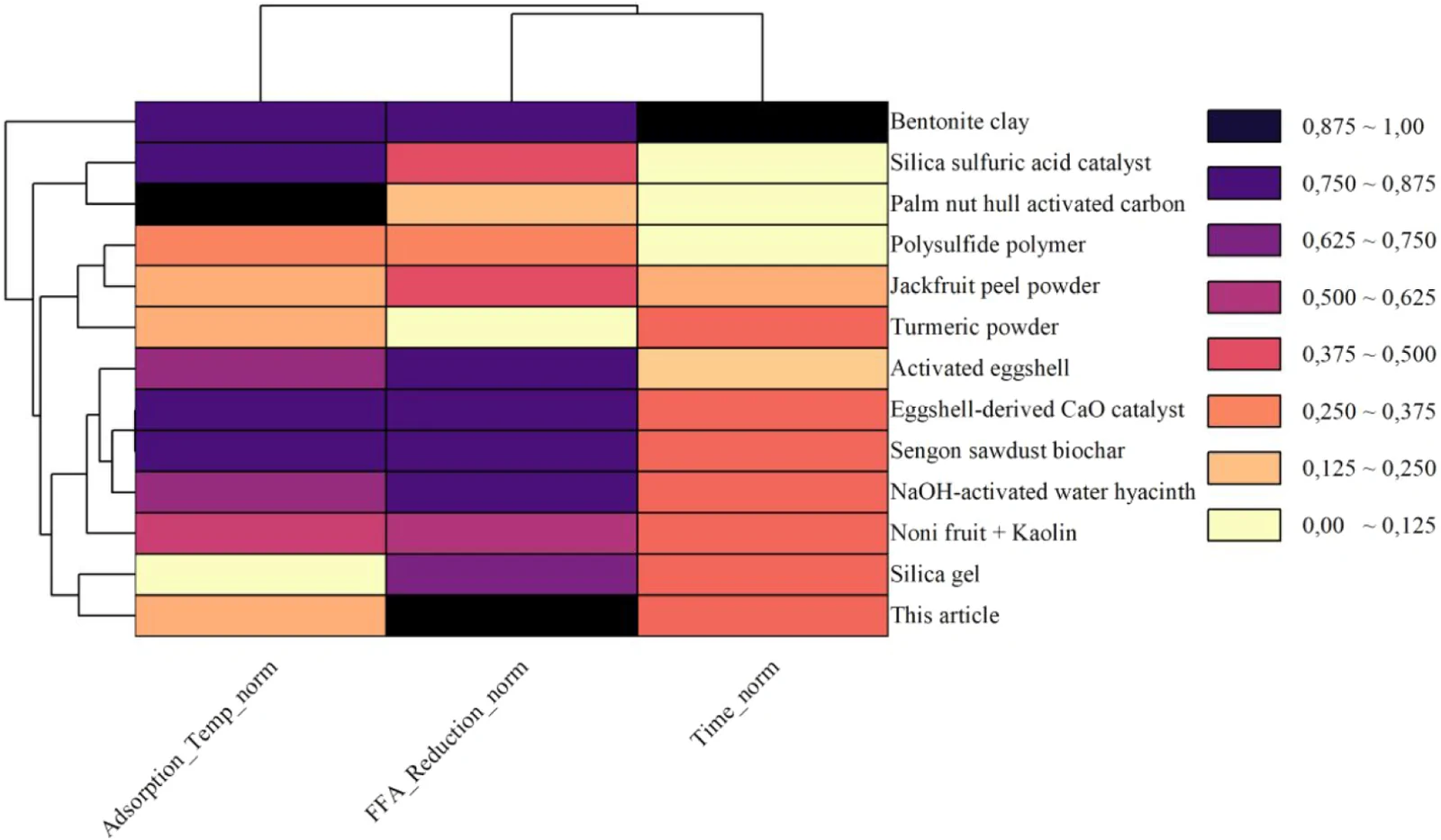
Comparison of adsorption parameters (temperature, free fatty acid reduction, and time) for different adsorbents.

Notably, the material investigated in this study clustered separately from most reported adsorbents, combining the highest normalized FFA reduction with comparatively low temperature and moderate contact time requirements. This performance highlights a favorable balance between adsorption efficiency and process mildness, which is rarely achieved simultaneously in bio-based or mineral adsorbents reported in the literature. Similar studies typically emphasize either high removal efficiency under severe conditions or moderate efficiency under mild conditions.^28–30^

## 4 Conclusion

This study confirmed that thermally treated concrete waste can serve as a highly efficient and sustainable adsorbent for the reduction of free fatty acids (FFAs) in waste cooking oil (WCO), providing a viable pretreatment route for biodiesel production. The calcination process at 850 °C significantly enhanced the alkaline reactivity and surface porosity of the material due to the formation of CaO and C–S–H phases, which promoted acid–base neutralization through chemisorption mechanisms.

The Elovich kinetic model (R^2^ = 0.9865) provided the best fit for the adsorption data, confirming that the process is governed by heterogeneous chemisorption. Under optimal conditions, the material achieved a significant reduction in acid value and an adsorption capacity of 75 mg g^–1^, comparable to conventional alkaline adsorbents such as quicklime or calcium silicate. A pH increase from 7.5 to 10.5, along with the identification of calcium carboxylates through FTIR analysis, further validated the occurrence of chemical adsorption and acid neutralization. While this reduction marks considerable progress, it is important to note that, depending on the initial acid value, further processing or prolonged contact time may be necessary to meet ANP standards for direct alkaline transesterification.

Based on the initial acid value of 2.539 ± 0.31 mg KOH g^–1^, the 60% reduction yields an estimated value of 1.016 mg KOH g^–1^. This remains above the ANP limit of 0.50 mg KOH g^–1^ for finished biodiesel (Resolution No. 920/2023), requiring a further 50.8% reduction through a second adsorption stage or acid-catalyzed esterification before transesterification.

From an environmental standpoint, the reuse of construction and demolition waste (CDW) as a reactive adsorbent contributes to waste valorization, resource recovery, and decarbonization of the biofuel sector. This approach reducing landfill burden while improving the sustainability of biodiesel production chains.
